# Supplementary material for: Changing cognitive chimera states in human brain networks with age: Variations in cognitive integration and segregation
Source: PLoS Comput Biol. 2025 Sep 2;21(9):e1013093. doi: 10.1371/journal.pcbi.1013093 (PMC12422585; doi:10.1371/journal.pcbi.1013093)
Supplement: S1 Appendix — (PDF) [file pcbi.1013093.s001.pdf]

S1 Appendix. Additional Tables and Figures.

Table A. Cognitive system assignments for each brain region.

Brain Region	Cognitive System
Lateral Orbitofrontal	Attention
Superior Parietal	Attention
Superior Temporal	Auditory
Transverse Temporal	Auditory
Caudal Anterior Cingulate	Cingulo-Opercular
Pars Opercularis	Cingulo-Opercular
Pars Orbitalis	Cingulo-Opercular
Rostral Anterior Cingulate	Cingulo-Opercular
Rostral Midde Frontal	Cingulo-Opercular
Supramarginal	Cingulo-Opercular
Caudal Middle Frontal	Fronto-Parietal
Inferior Parietal	Fronto-Parietal
Medial Orbitofrontal	Fronto-Parietal
Pars Triangularis	Fronto-Parietal
Frontal Pole	Fronto-Parietal
Insula	Fronto-Parietal
Isthmus Cingulate	Default Mode
Posterior Cingulate	Default Mode
Precuneus	Default Mode
Superior Frontal	Default Mode
Paracentral	Motor-Sensory
Postcentral	Motor-Sensory
Precentral	Motor-Sensory
Thalamus	Subcortical
Caudate	Subcortical
Putamen	Subcortical
Pallidum	Subcortical
Hippocampus	Subcortical
Amygdala	Subcortical
Nucleus Accumbens	Subcortical
BankSSTS	Ventral-Temporal
Entorhinal	Ventral-Temporal
Fusiform	Ventral-Temporal
Inferior Temporal	Ventral-Temporal
Middle Temporal	Ventral-Temporal
Parahippocampal	Ventral-Temporal
Temporal Pole	Ventral-Temporal
Cuneus	Visual
Lateral Occipital	Visual
Lingual	Visual
Pericalcarine	Visual

These cognitive system assignments correspond to both the left and right hemispheres of the brain, as well as the fine-grained atlas, containing the same brain region name. For example, the brain region 'L insula 1' and 'L insula 2' belong to the brain region 'insula' in this table and thus to same cognitive system.

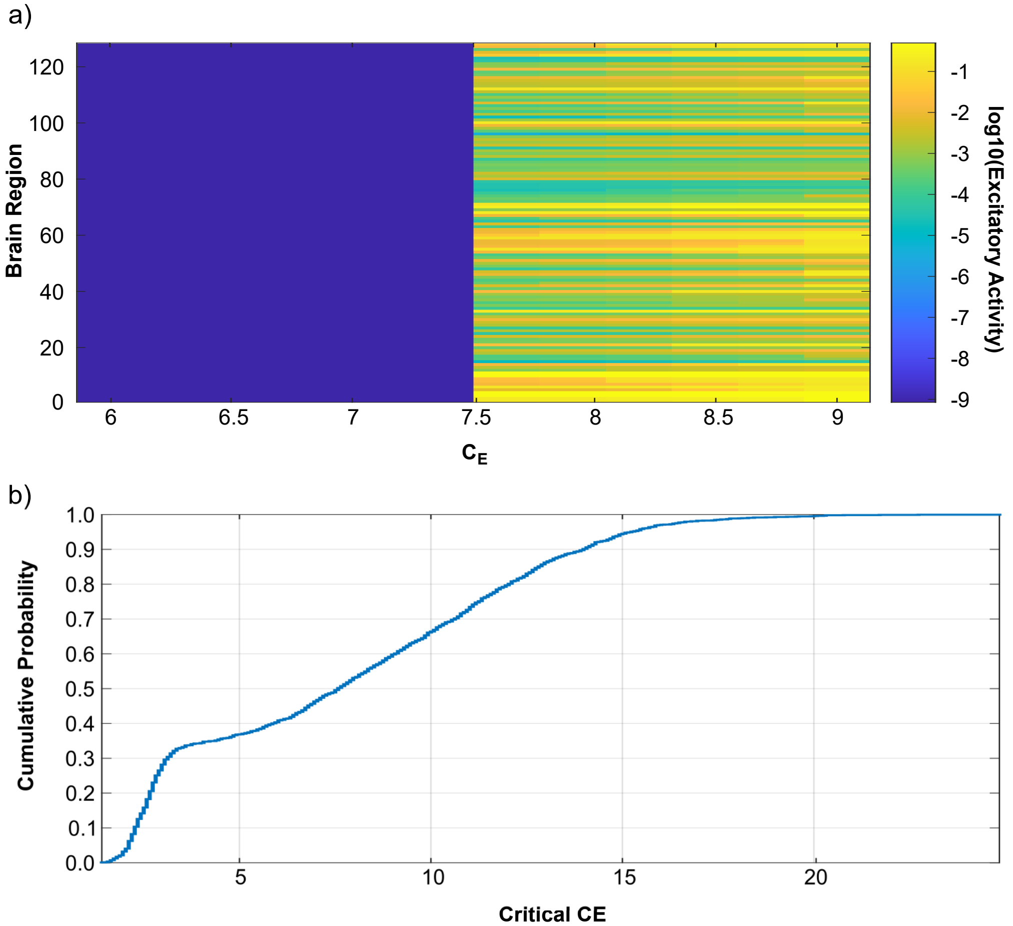

Fig A. a) Example of excitatory activity for each brain region as a function of C_E. b) Cumulative distribution function of the critical values of C_E for all $N = 2018$ structural connectomes.

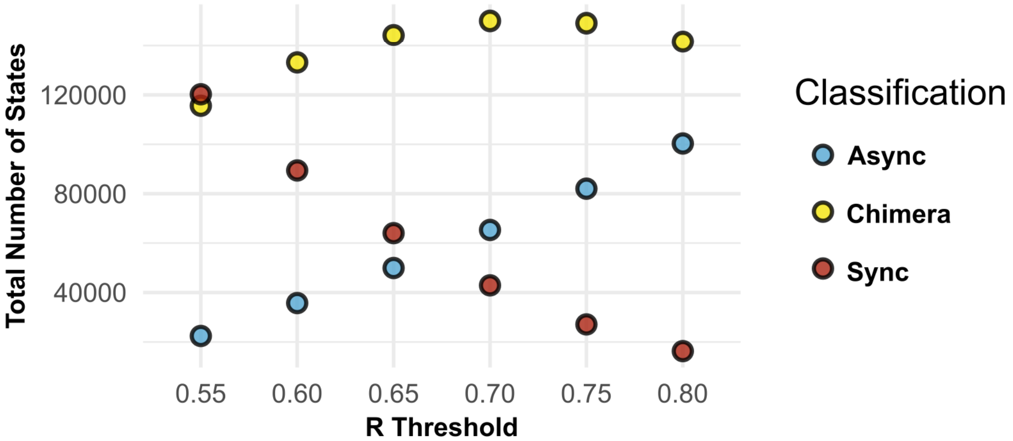

Fig B. The total number of synchrony classification states produces at different synchrony thresholds for the asynchronous states (async, blue), chimera states (yellow), and synchronous states (sync, red). We select the threshold at $R \geq 0.65$ for our analysis.

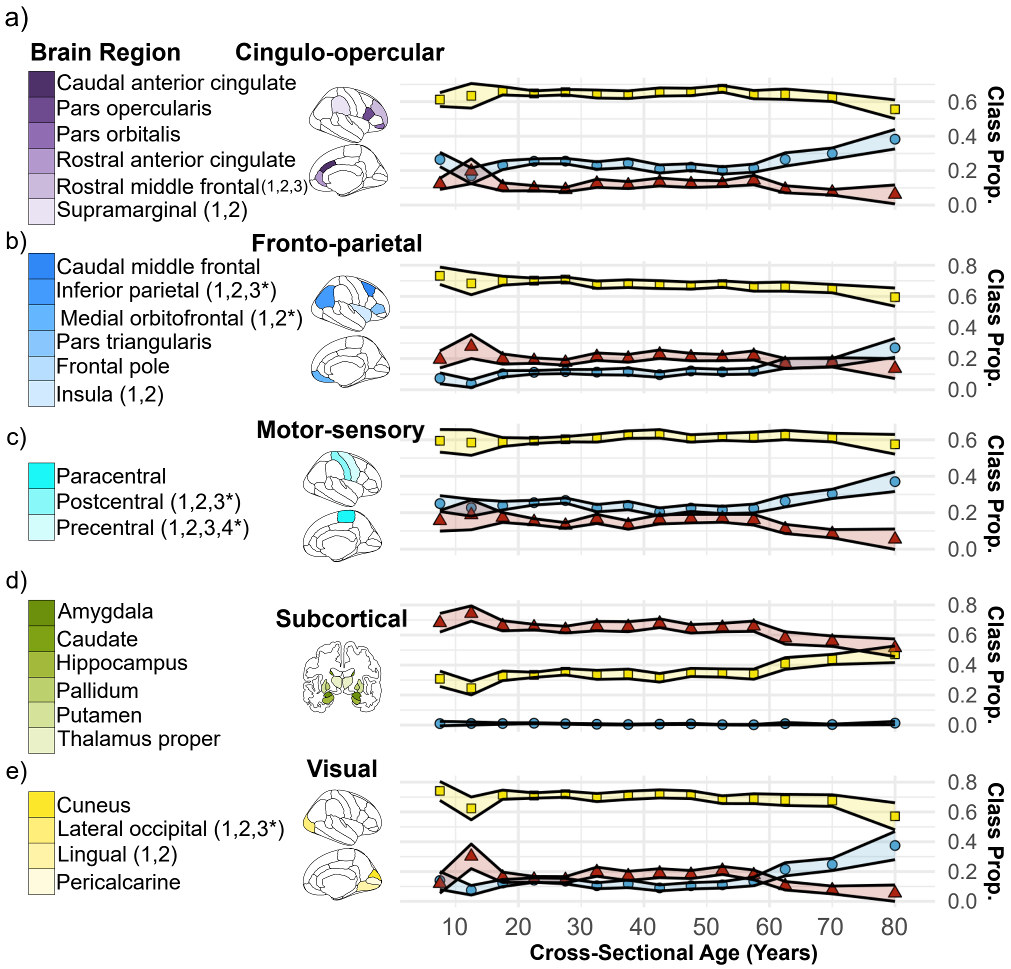

Fig C. The classification proportions across cross-sectional age for the following cognitive systems: a) cingulo-opercular (purple), b) fronto-parietal (blue), c) motor-sensory (cyan), d) subcortical (green), and e) visual (yellow). Blue circles represent the asynchronous classification proportions, yellow squares represent the chimera state classification proportions, and red triangles represent the synchronous classification proportions. The first column shows the brain region labels that belong to the respective cognitive system for each cognitive system, the second column shows where the brain regions are located spatially, and the third column shows how the classification proportions change with cross-sectional age. Shaded regions are 95% confidence intervals across individuals. *The number of subregions belonging to a brain region may differ depending on the hemisphere. In this case, the left-inferior parietal has 2 and the right-inferior parietal has 3, the left-medial orbitofrontal has 1 and the right-medial orbitofrontal has 2, the left-postcentral has 3 and the right-postcentral has 2, the left-precentral has 4 and the right-precentral has 3, and the left-lateral occipital 2 and the right-lateral occipital has 3. The maps of brain regions were drawn using the open-sourced ‘ggseg‘ package in R [77].

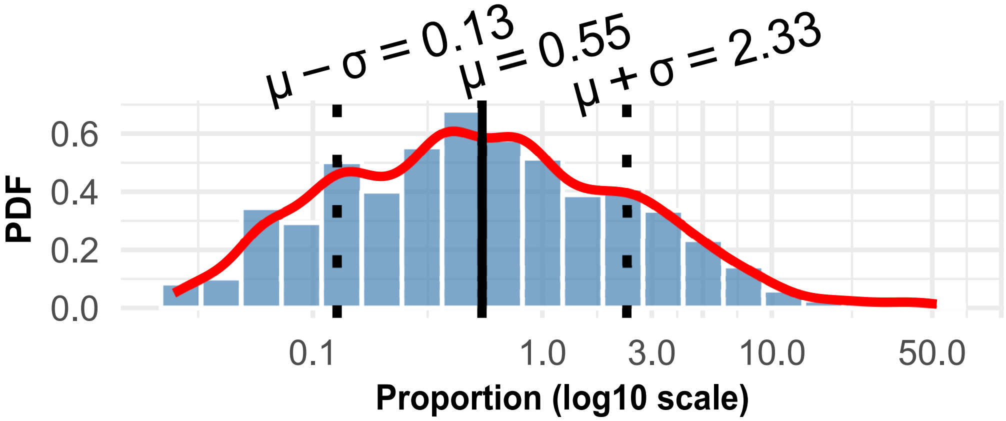

Fig D. The probability density function of all synchronization pattern proportions in log 10 scale with the mean μ (solid black line) and standard deviation σ (dotted black line). A kernel density estimator is shown in red.

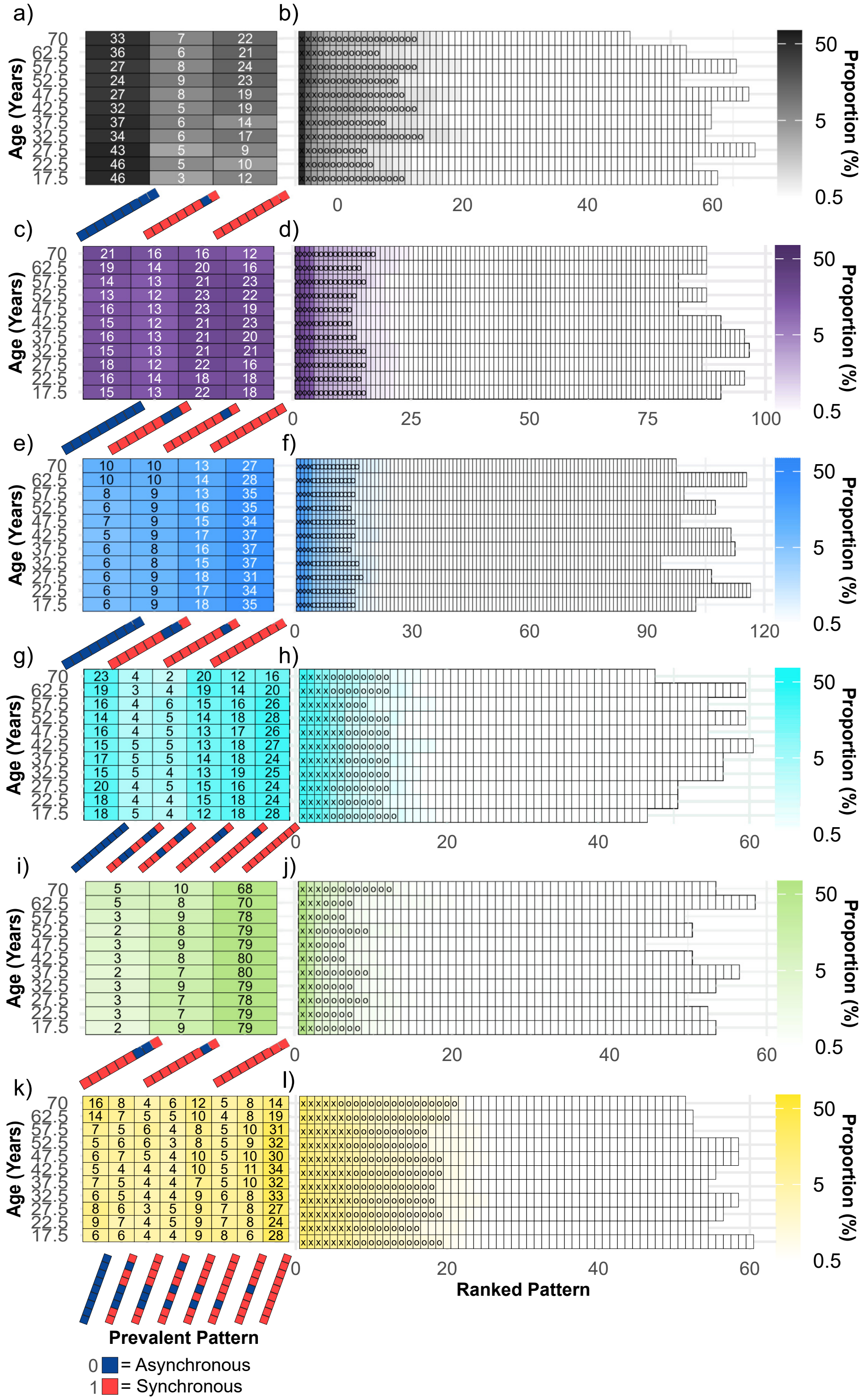

Fig E. Same as Fig 8 but for the auditory (panels a-b; grey), cingulo-opercular (panels c-d; purple), fronto-parietal (panels e-f; blue), motor-sensory (panels g-h; cyan), subcortical (panels i-j; green), and visual (panels k-l; yellow) cognitive systems.

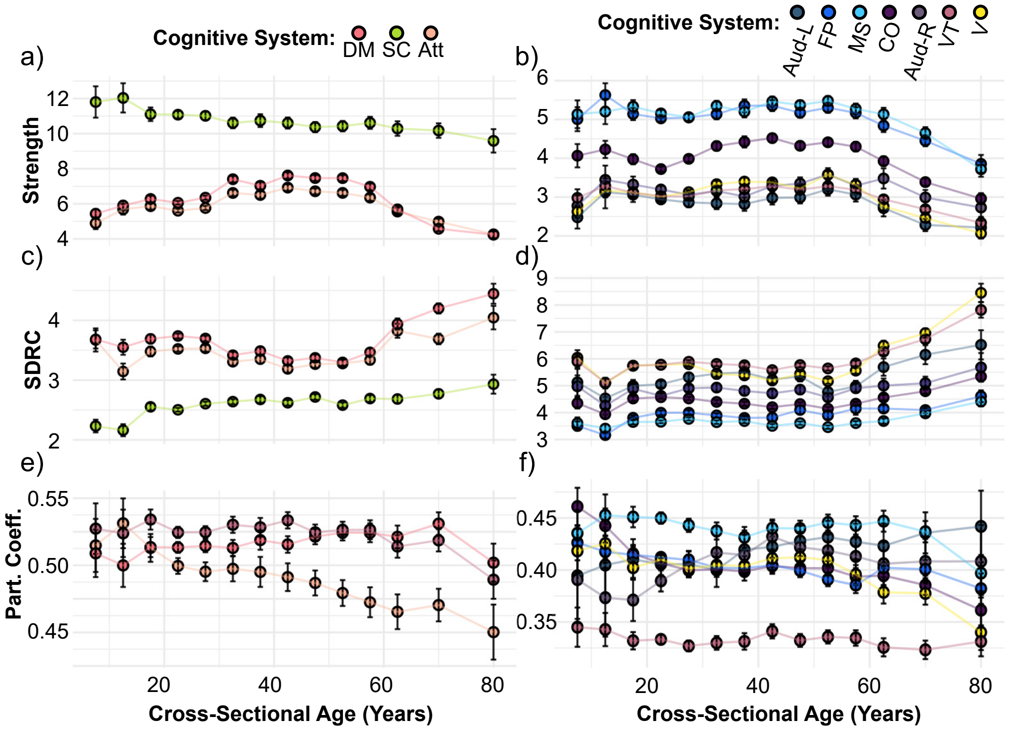

Fig F. Topological measures of the structural connectomes over cross-sectional age: The average strength (panels a and b), the shortest-distance to rich-club (SDRC; panels c and d), and the participation coefficient (Part. Coef.; panels e and f). Each color represents a different cognitive system, for panels a, c, and e orange for attention, pink for default mode, and green for subcortical and for panels b, d, and f blue-grey for left-hemisphere auditory, dark purple for right-hemisphere auditory, purple for cingulo-opercular, blue for fronto-parietal, cyan for motor-sensory, dark magenta for ventral-temporal, and yellow for visual. Error bars correspond to the standard error of the mean at the 2σ-level.

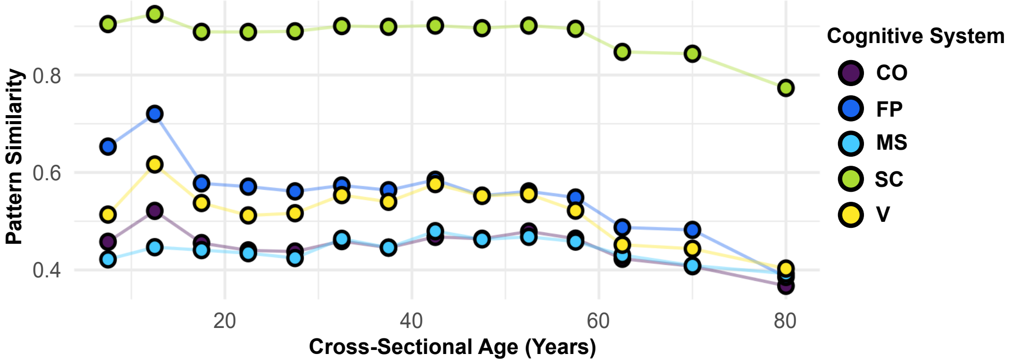

Fig G. Variations in similarity of synchronization patterns, as defined in Eq. (12), with cross-sectional age across the Cingulo-opercular (CO; purple), Fronto-parietal (FP; blue), Motor-sensory (MS; cyan), Subcortical (SC; green), and Visual (V; yellow) cognitive systems.

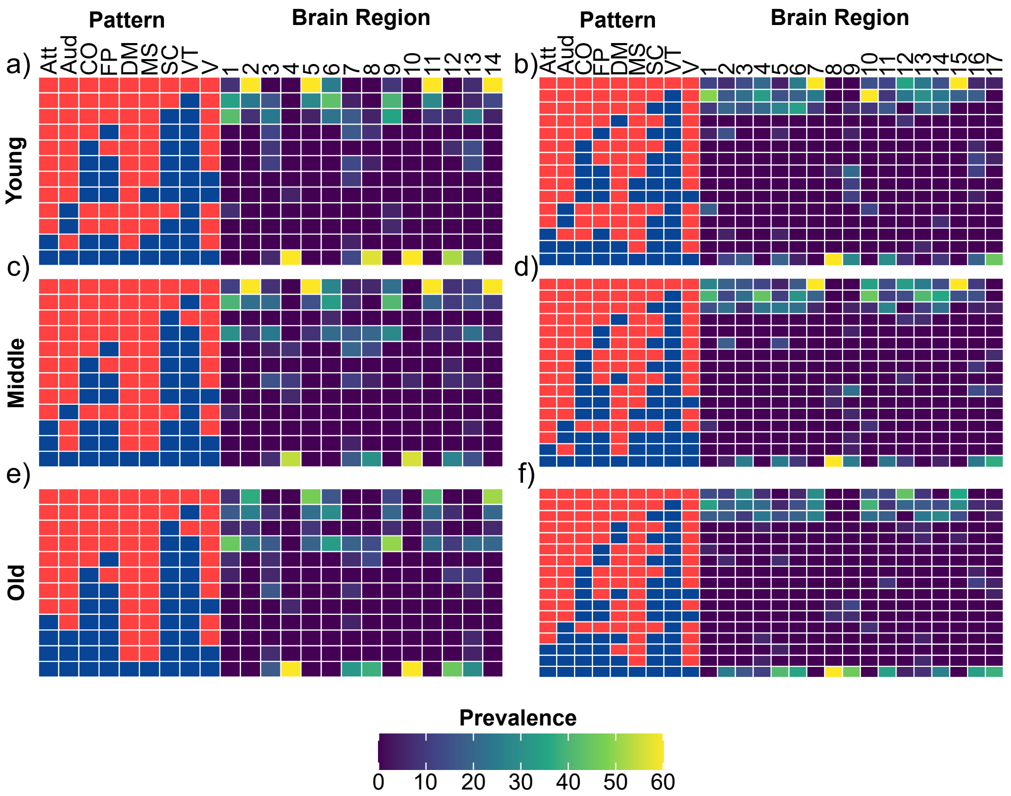

Fig H. The prevalence of synchronization patterns for each brain region belonging to the motor-sensory cognitive system (the first column, panels a,c,e) and cingulo-opercular cognitive system (the second column, panels b,d,f). The top (panels a,b), middle (panels c,d), and bottom row (panels e,f) represent the age group young (< 30 years), middle (≥ 30 & < 60 years), and old age (≥ 60 years), respectively. The columns titled with pattern represent the synchronization pattern and are colored red for synchronous and blue for asynchronous grouping with the left-to-right columns representing the attention (Att), auditory (Aud), cingulo-opercular (CO), fronto-parietal (FP), default mode (DM), motor-sensory (MS), subcortical (SC), ventral-temporal (VT), and visual (V) cognitive systems, respectively. The columns titled with brain region each represent a single brain region per column and are colored according to the prevalence of the emergent synchronization pattern when stimulating that brain region across all individuals in a given age group.

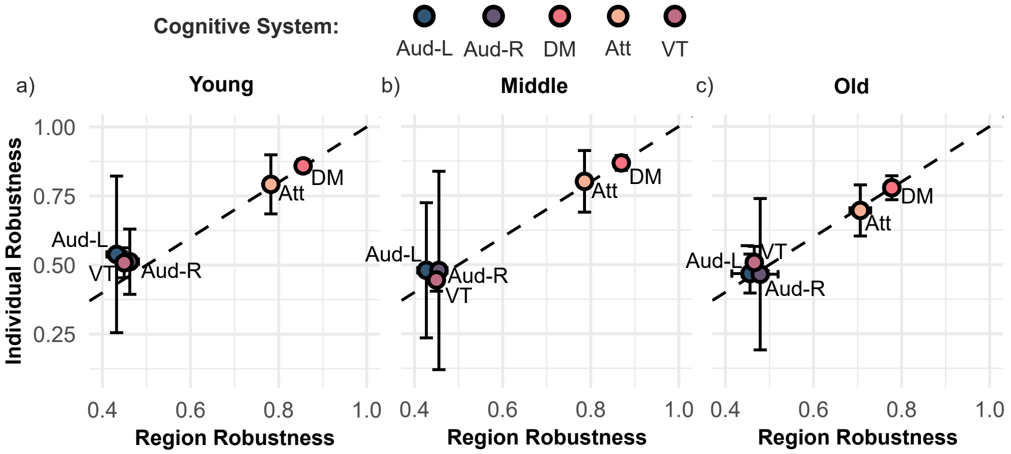

Fig I. Individual robustness and region robustness across the three age groups: a) young (< 30 years, b) middle-aged (≥ 30 & < 60 years), and c) old age (≥ 60 years) for five cognitive systems: auditory-left (Aud-L; dark blue), auditory-right (Aud-R; dark purple), ventral-temporal (VT; dark magenta), default mode (DM; pink), and attention (Att; orange).

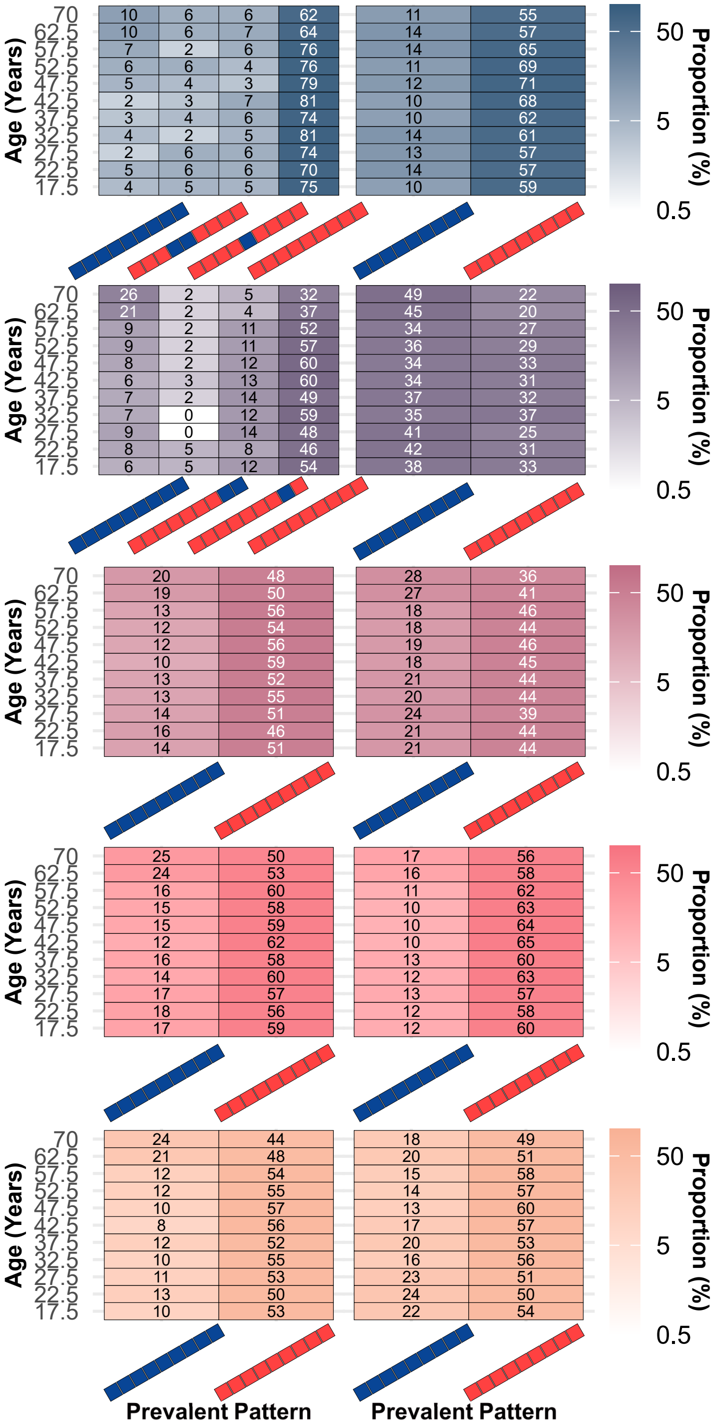

Fig J. Same as in Fig 8 but for randomized controls, where each brain region was randomly assigned to a cognitive system while keeping the number of brain regions for a given cognitive system fixed as in [53]: a) For a single random realization; b) For a set of ten different random realizations. In all cases, synchronous and asynchronous patterns dominate and chimera states are absent from the prevalent patterns, with the exception of cognitive systems with a small number of brain regions for a single realization. This is different from the results shown in Figs. 8 and E, where spatially distinct chimera patterns appear in the top two prevalent patterns in certain systems. This finding highlights the importance of uniting a cognitive systems framework with a chimera-based analysis to understand and interpret the emergent patterns of synchronization in the brain.
